# “Assistant physician light?” – Feedback from graduates on the practical year

**DOI:** 10.3205/zma001440

**Published:** 2021-02-15

**Authors:** Alexandra Eichhorst, Kevin Kunz, Marianne Giesler

**Affiliations:** 1 Albert-Ludwigs-Universität Freiburg, Medizinische Fakultät, Studiendekanat, Kompetenzzentrum Evaluation in der Medizin BW, Freiburg/Breisgau, Germany

**Keywords:** final year, medical education, workplace-based learning, qualitative analysis

## Abstract

**Background: **As the final and longest practical phase of undergraduate medical study in Germany, the final year is essential for the acquisition and development of core medical competencies. However, studies show that the educational conditions are often not optimal. The aim of this study was to learn more about the educational conditions connected with the final year and to find out how it can be improved. To do this, written comments from graduate evaluation surveys were analyzed.

**Methods:** Using the data from the survey of Freiburg medical students in the graduating classes of 2015/16 and 2016/17, we investigated which potential improvements were identified by students who had completed the final year and which aspects these students felt they especially benefited from in terms of beginning their medical careers. The written responses by the Freiburg graduating classes of 2015/16 (n=88; response rate: 28%) and 2016/17 (n=112; response rate: 36%) to the questions about beneficial aspects of the final year and potential improvements were qualitatively analyzed for content. As a comparison condition, the written comments of medical students graduating in the same years from the other medical schools in Baden-Württemberg were analyzed.

**Results: **The written responses of the Freiburg graduates to these two questions could be classified according to five main categories. Comments were most frequently assigned either to the category “(more) autonomous work, like an assistant physician” or “(increased) mentoring of the final-year students as learners”. In hindsight, the Freiburg medical graduates felt that they had already benefited in terms of beginning their careers from working independently under supervision during the final year, but they also saw room for improvement and wished that they had been perceived more strongly as learners and encouraged as such. The analysis of the written comments made by students in the same graduating classes at other medical schools in Baden-Württemberg showed corroborating results.

**Conclusion:** The results of this study show how the educational conditions of final year can be optimized. For instance, more opportunities should be created for final-year students to work independently and care for patients, and the course offerings should be expanded and adjusted, if needed, to match the needs of the students. Furthermore, those teaching final year students should be better trained and released from other duties so that they can focus on teaching.

## Zusammenfassung

**Zielsetzung: **Das Praktische Jahr (PJ) als letzter und zugleich längster Praxisabschnitt des Medizinstudiums in Deutschland ist entscheidend für den Erwerb und Ausbau ärztlicher Kernkompetenzen. Studien zeigen jedoch, dass die Ausbildungsbedingungen oftmals nicht optimal sind. Ziel der vorliegenden Studie war es, mehr über die Ausbildungsbedingungen im PJ zu erfahren, um ggfs. herauszufinden, wie das PJ verbessert werden kann. Hierzu wurden Freitextkommentare aus Absolventenbefragungen analysiert.

**Methodik: **Mit den Daten der Befragung von Freiburger Absolventinnen und Absolventen der Abschlussjahrgänge 2015/16 und 2016/17 wurde untersucht, welche Verbesserungsmöglichkeiten AbsolventInnen im Praktischen Jahr (PJ) sehen und von welchen Aspekten des PJ Absolventinnen und Absolventen der Humanmedizin bezüglich des Berufseinstiegs besonders profitieren konnten. Die Freitextkommentare zu den Fragen nach Verbesserungsmöglichkeiten sowie nach für den Berufseinstieg hilfreichen Aspekten des PJ der Freiburger Abschlussjahrgänge 2015/16 (N=88; Rücklauf 28%) und 2016/17 (N=112; Rücklauf 36%) wurden qualitativ-inhaltsanalytisch ausgewertet. Als Vergleichsbedingung wurden die Freitextkommentare von AbsolventInnen derselben Jahrgänge der anderen medizinischen Fakultäten Baden-Württemberg herangezogen.

**Ergebnisse: **Die Freitextkommentare der Freiburger Absolventinnen und Absolventen zu den beiden Fragen konnten jeweils 5 übergeordneten Kategorien zugeordnet werden. Am häufigsten ließen sich die Kommentare jeweils entweder der Kategorie „(Mehr) Selbstständiges Arbeiten wie ein/e Assistenzarzt/ärztin“ und „(Verstärkte) Förderung der PJ-Studierenden als Lernende“ einordnen. Rückblickend betrachtet, konnten Freiburger AbsolventInnen für den Berufseinstieg dementsprechend bereits von selbstständigem Arbeiten unter Supervision im PJ profitieren, sie sehen aber zugleich Raum für Verbesserungen des PJs und wünschen sich beispielsweise, verstärkt als Lernende wahrgenommen und gefördert zu werden. Die Auswertungen der Freitextkommentare derselben Abschlussjahrgänge von Absolventinnen und Absolventen anderer Standorte aus Baden-Württemberg erbrachten Ergebnisse, die mit denen der Freiburger Absolventinnen und Absolventen übereinstimmen.

**Schlussfolgerung: **Die Ergebnisse dieser Studie zeigen auf, wie die Ausbildungsbedingungen im PJ optimiert werden können. Beispielsweise sollen mehr Möglichkeiten zu selbstständiger Arbeit und Patientenbetreuung für PJlerInnen geschaffen werden, das Lehrangebot erweitert und gegebenenfalls an die Bedürfnisse der PJ-Studierenden angepasst werden. Darüber hinaus sollten PJ-BetreuerInnen besser geschult und für die Lehrtätigkeit freigestellt werden.

## Main claims

- In retrospect, medical graduates benefited most from working independently and assuming responsibility “like an assistant physician” during the final year of study.
- Medical graduates want to be perceived as learners even in the final year and to be mentored and encouraged in that role; they desire more practice-based instruction.
- At the same time, graduates want future final-year students to be given more opportunity to work independently and to assume responsibility for their work like an assistant physician would.
- These findings concretely show which expectations young physicians have when optimizing skills acquisition during the final year and can, as a result, contribute to the development of new measures and the expansion of existing ones to improve skills acquisition during the final year (teacher training, interprofessional training wards, training and simulation centers).

## Introduction

The final year is the longest practical phase of undergraduate medical study and represents a crucial educational stage for attaining the objectives defined in 2015 by the Medizinische Fakultätentag (MFT) and the German Association for Medical Education (GMA) in the National Competency-based Catalogue of Learning Objectives for Undergraduate Medical Education (NKLM), according to which competency-based medical study in Germany is organized. The final year, as described by the German medical licensure act (Approbationsordnung für Ärzte, ÄApprO, 02/08/2013), is focused on patient-based instruction in which students are expected to expand and deepen the medical knowledge and skills they have acquired in earlier semesters. Students are to learn to apply this expertise to specific medical cases. To accomplish this, teaching physicians should assign them medical tasks appropriate to their level of proficiency.

It is evident from the few previous studies that final-year students are not always satisfied with the conditions they experience. As shown by the results of a survey conducted by the Marburger Bund [1], many final-year students often work up to 50 hours per week and frequently perform nonmedical tasks during this time. On top of this, some did not feel properly mentored. One reason for this is the heavy workload experienced by the teaching physicians who have hardly enough time to give adequate instruction. These results can also be found in other studies [2], [3]. The basic conditions are also criticized in the 2018 online petition put forward by the BvMD (national medical student council) for “a fair practical year” [4]. This petition calls for improvement in the teaching and learning that take place in the final year with a set schedule of minimum weekly hours for teaching and self-directed study.

Given this context, the aim of this study was to learn more about which educational conditions in the final year medical graduates view as having helped prepare them well for entry into their profession and which improvements they recommend. Data from the graduate surveys were analyzed for this purpose. These surveys represent a valuable method for verifying, and if needed improving, the quality and general conditions of medical education [5].

## Methods

### Surveys

The surveys of the Freiburg University medical graduates, done as part of the BMBF project MER*LIN*, were carried out approximately one and a half years after graduation. Forty-nine questions address professional career, graduate satisfaction with their profession, retrospective evaluation of the study program, and a self-assessment of their competencies. The following open-ended questions, among others, were asked about the final year:

- Which aspects of the final year contributed in particular to you feeling prepared to pursue your profession?
- Which aspects of the final year would you change so that future physicians are even better prepared to take up the practice of medicine?

Data for the graduating classes of 2015/16 and 2016/17 were drawn upon to analyze the answers to the open-ended questions about the final year because these were the years in which the two questions were first included in the survey. The respondents had the option to participate by mail or online.

#### Sample

The data for the 2015/16 and 2016/17 graduating classes from all of the medical schools in Baden-Württemberg were included in the analysis. The results for the Freiburg University graduates, who voluntarily participated in the survey (N_FR_Jg.15/16_=88; N_FR_Jg.16/17_=112), were compared with the compiled results for the four other medical schools in Baden-Württemberg (N_VG_Jg.15/16_=293; N_VG_Jg.16/17_=224). The response rates were 29% and 36%, respectively. Basic characteristics of the surveyed Freiburg cohorts and the comparison group are presented according to year of graduation in table 1 (Tab. 1).

#### Qualitative analysis of the written responses

The written responses to the questions about potential improvements and the helpful aspects of the final year regarding entry into the medical profession collected from the 2015/2016 und 2016/2017 graduating classes from Freiburg Medical School were categorized and qualitatively analyzed for content according to Mayring [6]. In the course of doing this, main categories were inductively identified from the responses; these categories summarize the central themes and structure the data. In doing this, the individual responses were consolidated into typologies about which generalizable, meaning quantitative, statements could be made about frequency distributions. When compared to data in other categories, the data of a particular category shows the least difference possible within the category and the most difference possible outside the category.

## Results

### Category formation using qualitative content analysis

The results of the qualitative content analysis encompass the identified categories and the percental distribution of the statements to the categories and subcategories.

A total of 122 out of the 200 Freiburg graduates participating in the survey responded with written comments to question 1, *Which aspects of the final year contributed in particular to you feeling prepared to pursue your profession?*

Five main categories were formed based on the dataset:

- *Working autonomously, like an assistant physician: *The graduates’ statements make it clear that they experienced the greatest gain in competence when they could work independently, taking responsibility under competent supervision. An example comment is: “attend my own patients and then discuss the cases with an experienced assistant physician or senior physician”.
- *Being perceived as a learner and being mentored accordingly: *Comments which highlighted teaching and/or supervising in the final year as beneficial for acquiring occupation-specific competence were compiled in this category. For example, “a lot of clinical work, a lot of interaction with patients, seminars taken in parallel to deepen the clinically acquired knowledge”.
- *Structure and organization of the final year: *Comments by graduates about a successful final year are assigned to this category (e.g. satisfaction with the option to elect a subject). Other examples: are “rotations through different departments, dedicated assistant physicians”.
- *Little/Nothing: *All graduate comments evaluating the final year as a mostly negative experience that did nothing to prepare them for professional medical practice are summarized in this category. For example, “Overall, the final year was not really helpful for me, basically a waste of time”.
- *Miscellaneous: *This category serves to capture all graduate comments that could not be clearly assigned to any of the other main categories or their subcategories. For example, an offer of a job after the final year.

A total of 103 out of the 200 Freiburg graduates responded with comments to question 2, *Which aspects of the final year would you change so that future physicians are even better prepared to take up the practice of medicine?*

These comments were assigned to four main categories that are for the most part similar to those for question 1:

- *Working more autonomously, like an assistant physician: *All of the statements by graduates that express not having had enough latitude in the final year to do independent work for which responsibility is taken (as a physician would) are assigned to this category; often with the simultaneous indication that working autonomously and taking responsibility is an optimal way to acquire competence during the final year. For example, “more opportunity to independently provide patient care” and “assuming the main tasks of a physician in the daily routine under constant supervision”.
- *Stronger perception of final-year students in their role as learners and stronger encouragement of them in that capacity: *The graduates who commented on this category expressed a desire to be seen more clearly as learners, e.g. through more practice-based instruction during the final year. For example, “putting final-year students to use as learners and not just as available work staff (particularly in internal medicine and surgery)”.
- *Improve the structure and organization of the final year:* Comments about obstacles to a successful final year, the feedback process, and suggestions for improving the organizational structure were gathered together in this category. For example, “appropriate compensation, position, and respect for practical-year students in the medical team; self-determined organization of the trimester (e.g. students interested in surgery could spend 4 months in surgery and only 2 months in internal medicine, and so on)”.
- *Miscellaneous: *This category captures all comments made by graduates that could not be clearly assigned to any of the other categories. For example, “fair and equivalent oral exams following the final year”.

Based on the content analysis, 13 subcategories were identified based on the responses to question 1 about the positive aspects of the final year. From the responses to question 2 about potential improvements, 17 subcategories became evident to which the responses were assigned. These subcategories, stemming from their respective main categories, are listed in table 2 (Tab. 2).

#### Distribution of the responses among the categories

Regarding the question which aspects of the final year helped with starting out in the medical profession (question 1), over half (n=117) of the graduates gave positive feedback. The percentage of graduates who made concrete suggestions for improvement (question 2) is approximately the same (n=103).

Since multiple answers were possible for each free text field, meaning the use of keywords which could be assigned to several (main) categories, the number of keywords mentioned exceeded the number of given responses. As a consequence, the percentages in the following statistical analysis always refer to the number of keywords mentioned for each category and not to the number of given responses. In table 3 (Tab. 3), the number of mentioned keywords and the number of given responses for both questions are differentiated for Freiburg and the comparison group according to graduation year.

**Figure 1:**
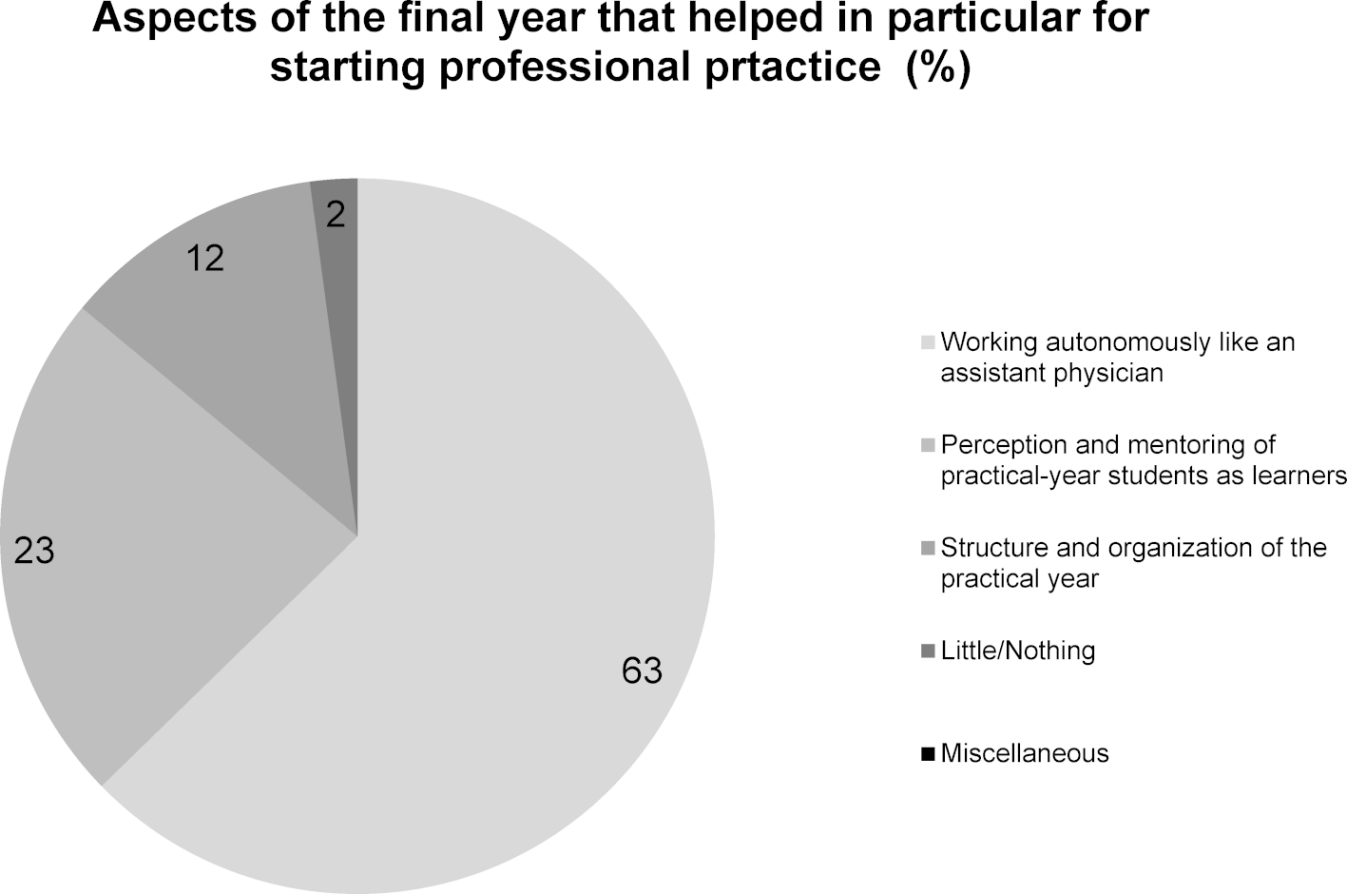
Responses to question 1: Which aspects of the practical year contributed in particular to you feeling prepared to pursue your profession?

**Figure 2:**
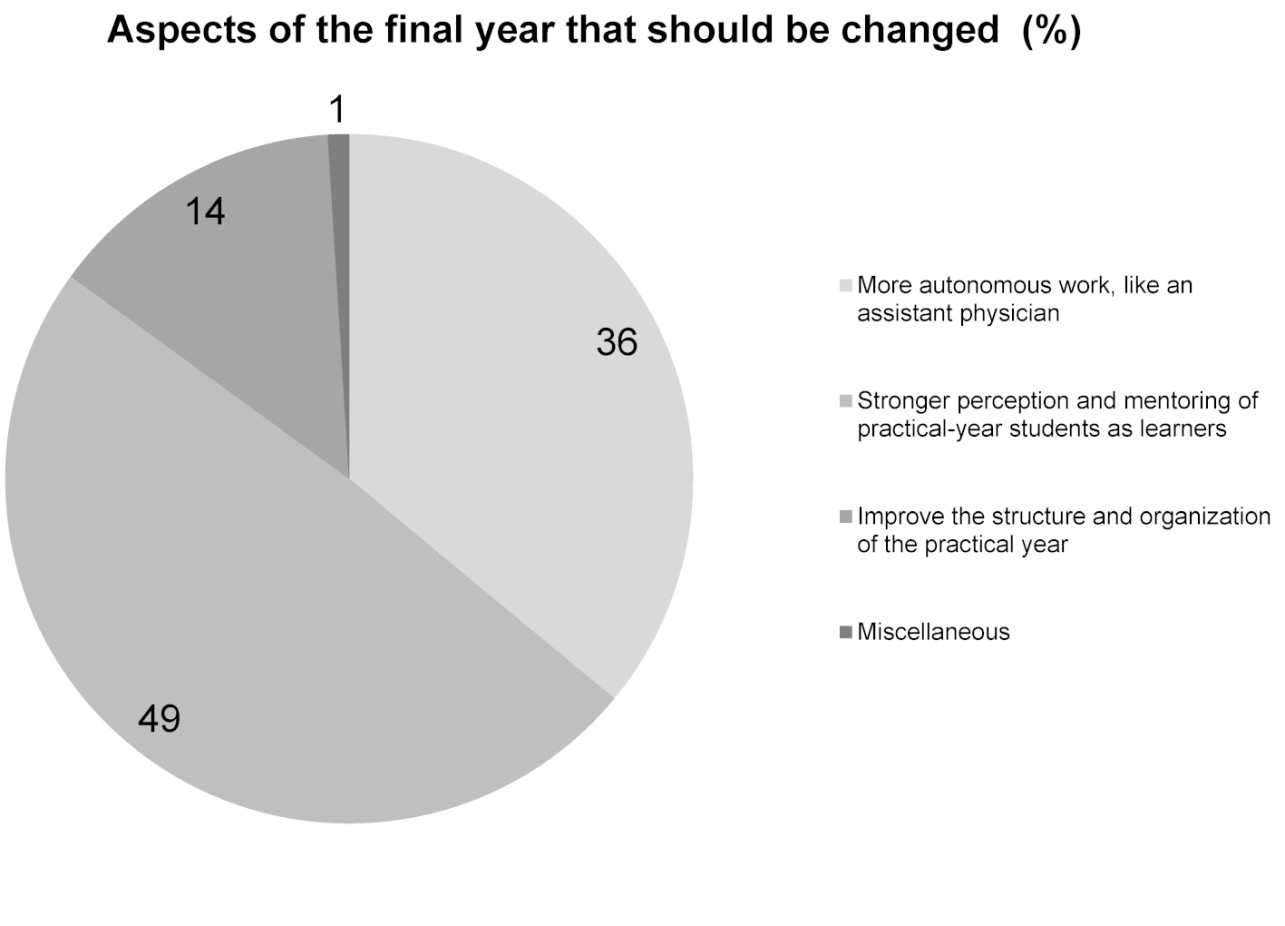
Responses to questions 2: Which aspects of the practical year would you change so that future physicians are even better prepared to take up the practice of medicine?

In response to the question which aspects of the final year helped in particular for starting professional practice (question 1), 148 of 236 mentions (63%) – the most answers relatively viewed – fell into the category working autonomously like an assistant physician (see figure 1 (Fig. 1)). Of the responses to the question about potential improvements to the practical year (question 2), about half of the written comments (99 of 204 mentions) were assigned to the category perceive final-year students more strongly as learners and mentor them accordingly. With 36%, the second most frequent mentions indicated a wish for the ability to work more independently during the final year, like an assistant physician would (see figure 2 (Fig. 2)). A total of 12% of the mentions in response to the question about the positive aspects of the final year (question 1) fell into the category structure and organization of the final year, (see figure 1 (Fig. 1)) and 14% of the keywords mentioned to the question about potential improvement (question 2) into the category improve the structure and organization of the final year (see figure 2 (Fig. 2)). Only 2% of the graduates responded to the question about which aspects were particularly helpful for beginning their professional practice with “none” or “few” (see figure 1 (Fig. 1)).

**Figure 3:**
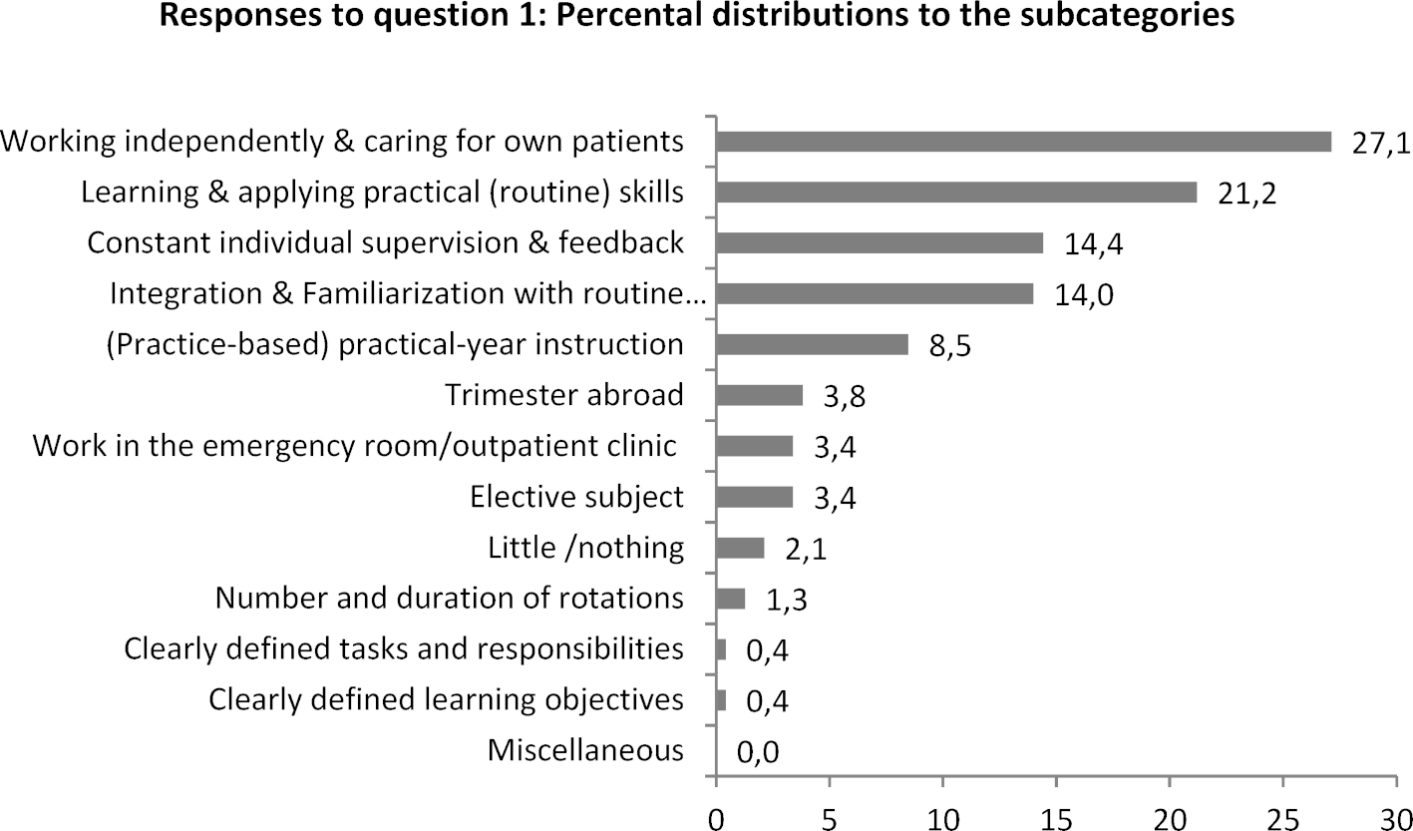
Percental distribution of the mentions to the subcategories for question 1: Which aspects of the practical year contributed in particular to you feeling prepared to pursue your profession?

**Figure 4:**
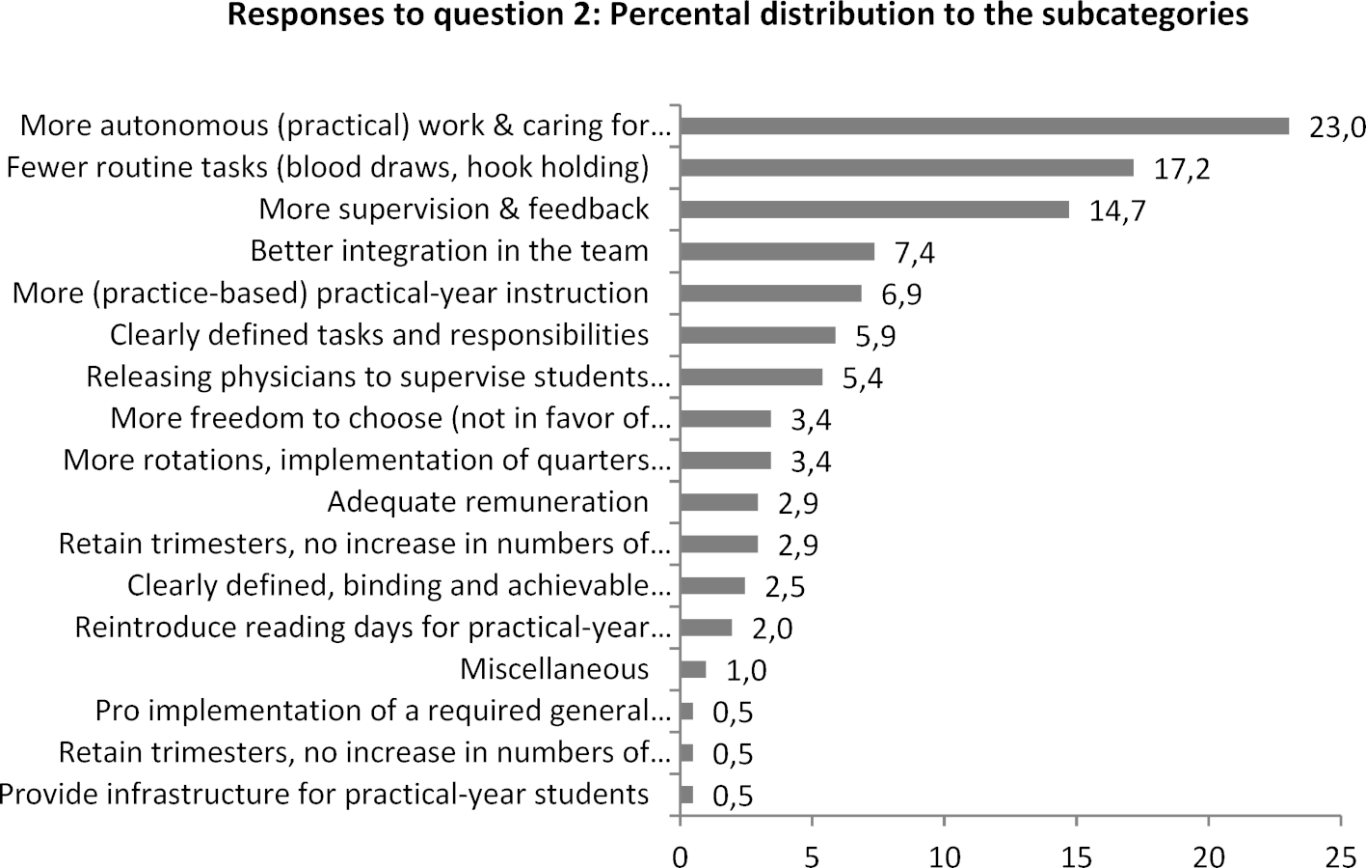
Percental distribution of the mentions to the subcategories for question 2: Which aspects of the practical year would you change so that future physicians are even better prepared to take up the practice of medicine?

The distribution of the responses (number of keywords mentioned) among the separate subcategories is presented in figure 3 (Fig. 3) and figure 4 (Fig. 4). It is notable that, relatively viewed, the most responses to both questions are placed in the subcategory (more) independent work and (stronger) perception of final-year students as learners and mentoring them accordingly. While it was stated that working autonomously did contribute in particular to being prepared for future medical practice, what graduates primarily wish for future final-year students is that they are perceived more strongly as learners. Especially frequent were the wishes that being assigned routine tasks, such as drawing blood, would come at less of a cost to clinical training and, overall, students be more intensively and constantly mentored and supervised by responsible teaching physicians.

Analysis of the keywords mentioned by the comparison group for both of the questions confirmed the results of the Freiburg cohorts to the greatest extent possible (see table 4 (Tab. 4)).

## Discussion

The objective of this study was to find out more about the educational conditions of the final year and possibly to find points for improvement. To accomplish this, the written responses to two open-ended questions on the graduate survey specifically about the final year were analyzed in terms of content. One question referred to aspects that had helped with starting out in the medical profession while the other asked for concrete suggestions that could help improve the final year.

The results show that graduates hope that future final-year students will be able to work more independently under supervision. Like a “light version of an assistant physician”, according to one female respondent. At the same time, in keeping with this, working independently and taking responsibility for the care of one’s own patients during the final year are retrospectively evaluated as being especially valuable for gaining competence and subsequently beginning a medical career. The desire to develop and expand core medical skills within the scope of the final year through increased independent, supervised patient management has already been reported in two qualitative studies of final-year students at two medical schools in Baden-Württemberg [2], [7] and a small cohort of students from the final year and the block practicum in general practice [3]. In their study, Schrauth et al. [2] report findings that align with our results showing that graduates retrospectively criticize not being adequately perceived as learners and mentored in that capacity during the final year. According to that study, final-year students particularly criticize the lack of time their supervising physicians spent, accompanied by the lack of feedback and teaching and too much time spent on routine work. Similar complaints were reported in an interview study involving final-year students at Heidelberg [7]: Excessive deployment to perform routine tasks and a lack of integration into the team were cited by the students as a source of stress and an obstacle to professional development. Overall, the results of these studies support the findings here. It can be deduced from this that there is a need to develop measures to promote the opportunity for autonomous work to improve the acquisition of medical competence during the final year.

In recent years several projects have been initiated in Germany to improve quality and optimize the acquisition of competence during the final year: The desire of final-year students to be seen as learners has been responded to at the medical schools in Baden-Württemberg by the MER*LIN* project with the introduction of regularly scheduled training sessions for the educators who teach specifically in the final year and the distribution of a handbook for those educators [https://www.merlin-bw.de/publikationen/manual-fuer-pj-betreuer.html]. To help grant the graduates’ wish for more feedback from their final-year teachers, pocket-sized cards with feedback information and a handbook on how to give feedback were also developed in Freiburg for students and teachers as part of the MER*LIN* project. At the Friedrich Schiller University of Jena, as part of the PJ *Plus* program, the acquisition of core medical skills is supported through constant mentoring of the students [https://www.uniklinikum-jena.de/studiendekanat/PJPLUS.html]. Furthermore, pilot projects, such as Heidelberg’s HIPSTA interprofessional training ward in general and visceral surgery [8] and Freiburg’s pilot project IPAPÄD (Interprofessional pediatric training ward at the Medical Center for Pediatrics and adolescents Freiburg) [9], are enabling final-year students, in cooperation with nursing students, to independently organize and care for an entire hospital ward under the supervision of senior medical staff, thus allowing the practical-year students to work in a capacity similar to that of an assistant physician on an interprofessional team.

The curricular concept for surgery designed by Kadmon et al. [10] is forward-looking in terms of enabling students to work with more autonomy while under supervision. Applying the model of “entrustable professional activities” (EPA), students acquire professional skills during the final year through “units of professional practice,” defined as responsible tasks, which are assigned on the condition that the students have already demonstrated sufficient competence to perform them. To ensure patient safety and adhere to legal requirements, the EPAs in the final year usually involve activities classified as level 2 (allowed to perform under direct supervision) and level 3 (allowed to perform under indirect supervision). However, Kadmon et al. also point out that there are tasks which can be delegated in full during the final year, explicitly stating that tasks going beyond simple psychomotor skills and demanding time management, patient interaction and teamwork, up to EPA level 4 should be performed because the ability to perform these independently will be assumed upon beginning postgraduate medical training (pg. 6).

Most of the strategies and approaches described here to increase autonomy and individual responsibility, and thus improve skills in final-year students, demand the presence of motivated and trained medical educators. Yet, under the current conditions the responsible physicians are usually unable to fulfill their teaching duties due to their own high workloads. As Schrauth et al [2] emphasize in their study, radical and far-reaching political measures are necessary here to ensure that the institutional mandate imposed on university hospitals and academic teaching hospitals is carried out (pg.173).

### Special aspects and limitations of the study

It is noticeable in this study that the lack of supervision is not brought up as often as in the cited studies. Among other explanations, one reason for this could lie in the written form of the survey compared to the two cited studies in which focus group interviews were held. The wording of the two questions could also be responsible. The questions were intentionally formulated in the affirmative to elicit more substantial statements and avoid the usual brief responses about experiences in the final year, such as “more practice” and “better supervision.”

It must also be taken into account that, while the written responses came from the Freiburg graduates, they only allow limited conclusions to be drawn about the conditions of the final year in Freiburg. Our analyses show that the surveyed graduates did not complete all of their trimesters at hospitals affiliated with their university: 76% of the Freiburg cohort spent a part of the final year abroad. To verify the comparability of the data collected on the final year, graduate surveys at other medical schools in Baden-Württemberg were also analyzed according to the same hypothesis and method and then compared to the results for Freiburg. The same categories were identified. The results of the comparison group hardly deviate al all from the results seen for the Freiburg graduates (see table 4 (Tab. 4)) and indicate a high degree to which the Freiburg results can be generalized.

Regarding the validity of this study, it must be noted that its findings are the result of qualitative content analysis of written comments made by Freiburg medical graduates. A potential limitation of the analysis is the subjectivity involved in the identification of the categories. Since similar categories were also inductively formed with a high level of consensus by another medical collaborator and similar categories have also been identified in other qualitative studies focused on the final year [2], [3], [7], we still assume a certain generalizability of the results. Also, not all of the 200 participants in the two cohorts analyzed (representing only about a third of graduates) answered the open-ended questions. A skewing of the results as a result of selection bias cannot be ruled out because it is possible that the graduates who took the time to participate in the survey and write out answers to the open-ended questions do not necessarily represent the average members of the cohort. As a consequence, a fundamental skewing of the data cannot be ruled out which should be considered when thinking about generalization.

## Conclusion

The analysis shows that the Freiburg graduates, when they looked back at starting their professional careers, had been able to benefit from working autonomously under supervision during their final year of medical study. At the same time, the graduates saw room for improvement of the final year and wanted to be perceived more strongly as learners and mentored accordingly. These results could contribute to the development and expansion of measures to improve the acquisition of skills during the final year in that, for instance, more opportunities are created for final-year students to work independently and care for patients while being supervised and, at the same time, those who teach final-year students are directly strengthened in their role as educators and given appropriate teacher training. This would, however, also mean that medical educators responsible for teaching in the final year would need to be given more time for teaching duties.

## Funding

The graduate survey was carried out within the scope of the BMBF-funded MERLIN (Medical Education Research – Lehrforschung im Netz BW) project at the medical schools in Freiburg, Heidelberg, Mannheim, Ulm and Tübingen, under the leadership of Freiburg. The data and analysis presented in this paper come from the Freiburg office; funding code: 01Pl12011B.

## Ethics

This research was carried out in compliance with the Declaration of Helsinki and approved by the Ethics Commission at the Medical School of the Albert Ludwig University in Freiburg (446/16). All of the participants were informed in writing about the study and gave their consent. Participation was voluntary. No personal data of the participants have been reported in the study.

## Acknowledgement

We wish to thank our cooperative partners in the MERLIN project at the medical schools in Heidelberg, Mannheim, Tübingen and Ulm for their participation and productive collaboration in the graduate surveys. Special gratitude goes to the graduates themselves for participating in the survey.

## Competing interests

The authors declare that they have no competing interests.

## Figures and Tables

**Table 1:**
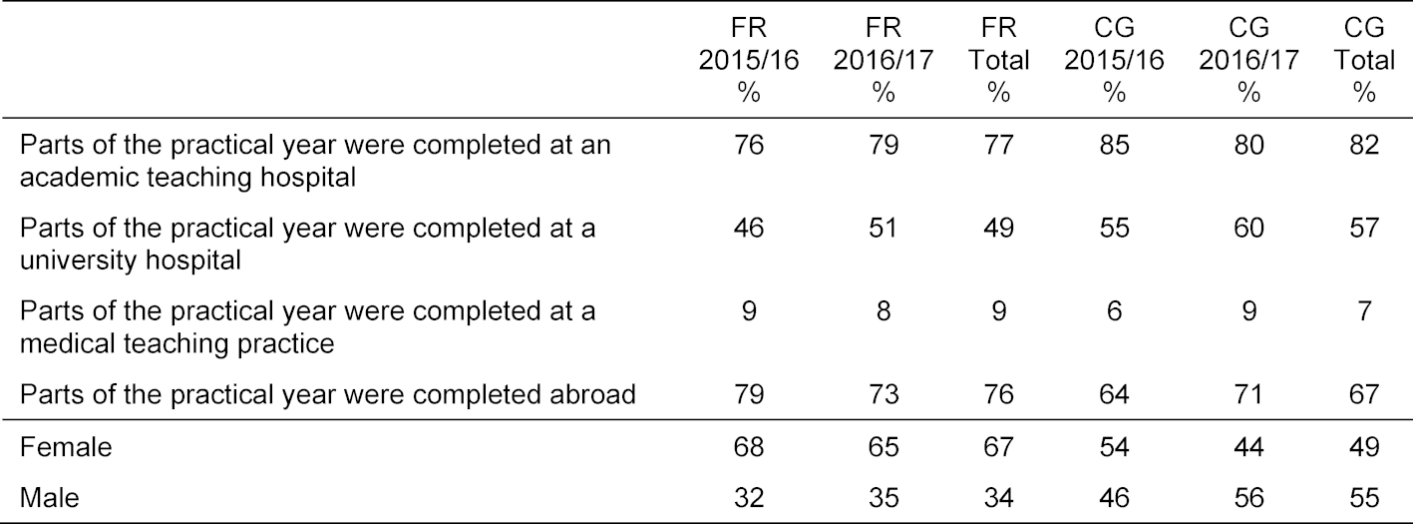
Data for the Freiburg sample (FR) and for the comparison group (CG), as percentages

**Table 2:**
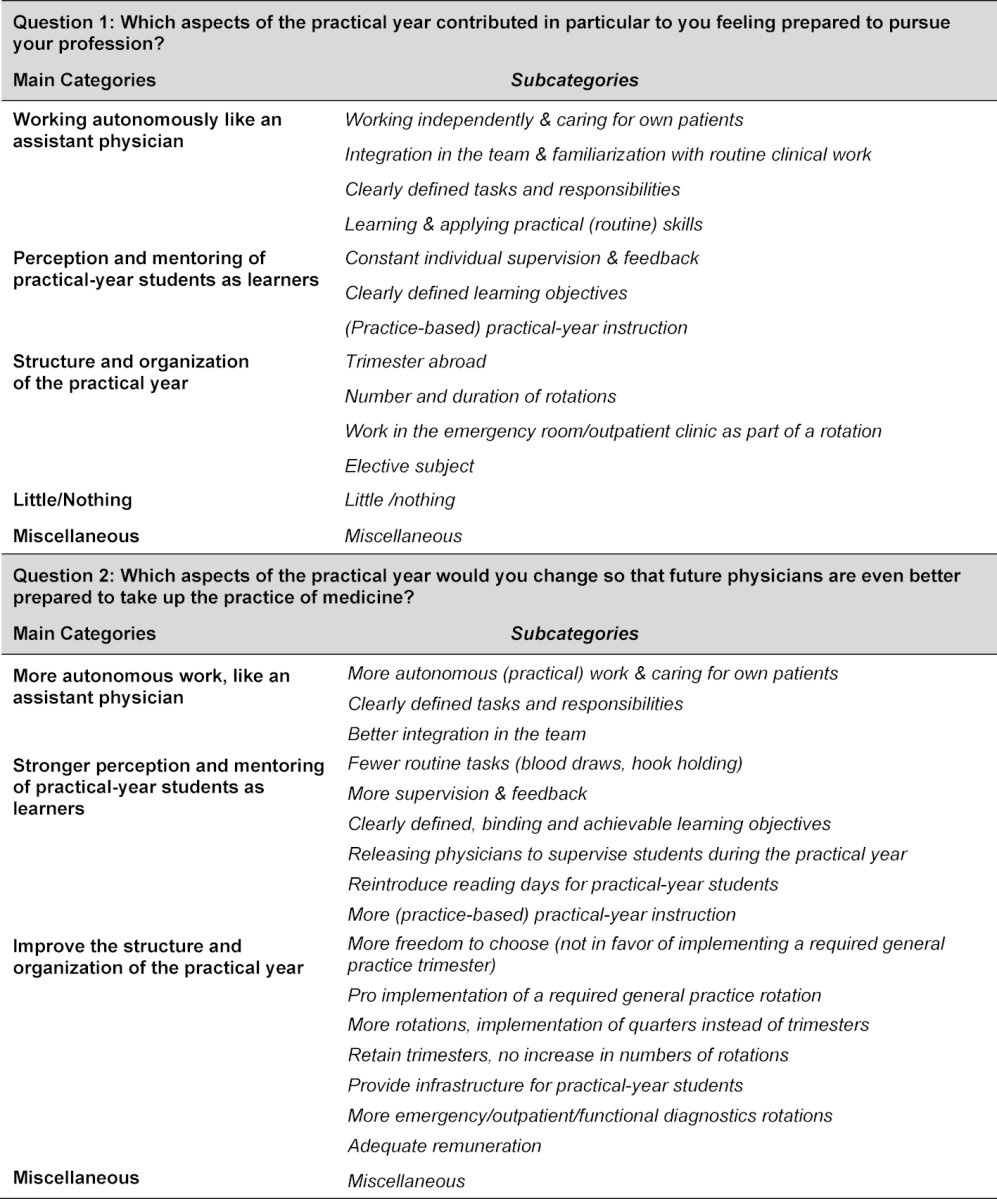
Categories and subcategories identified for questions 1 and 2

**Table 3:**
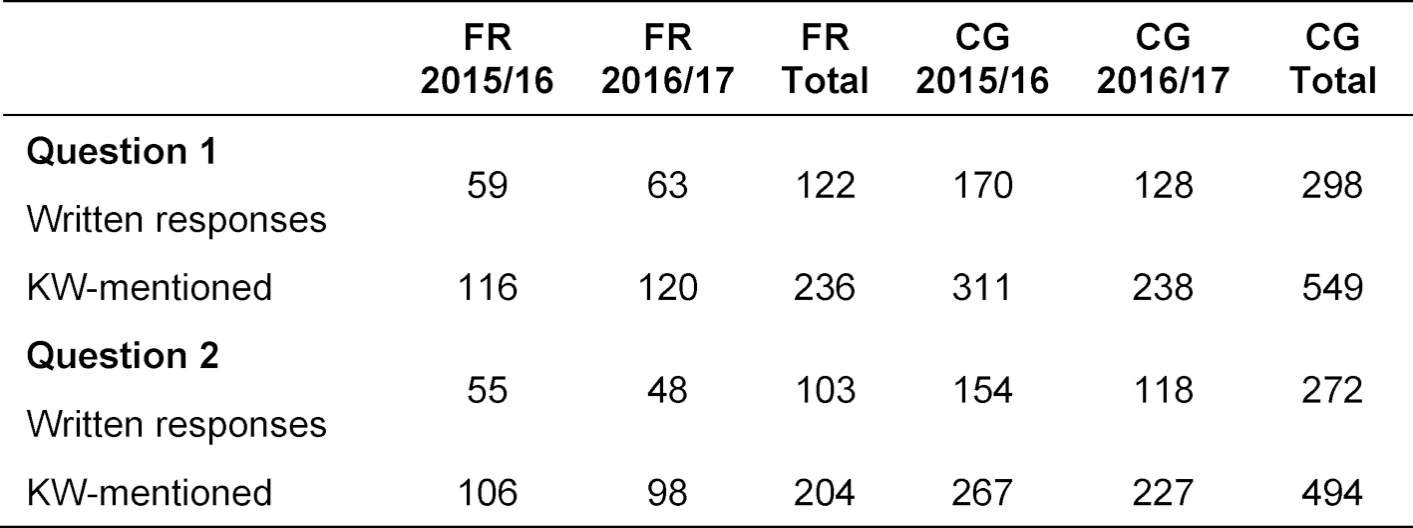
Number of written responses and number of keywords (KW) mentioned for Freiburg (FR) and the other medical schools in Baden-Württemberg (comparison group = CG)

**Table 4:**
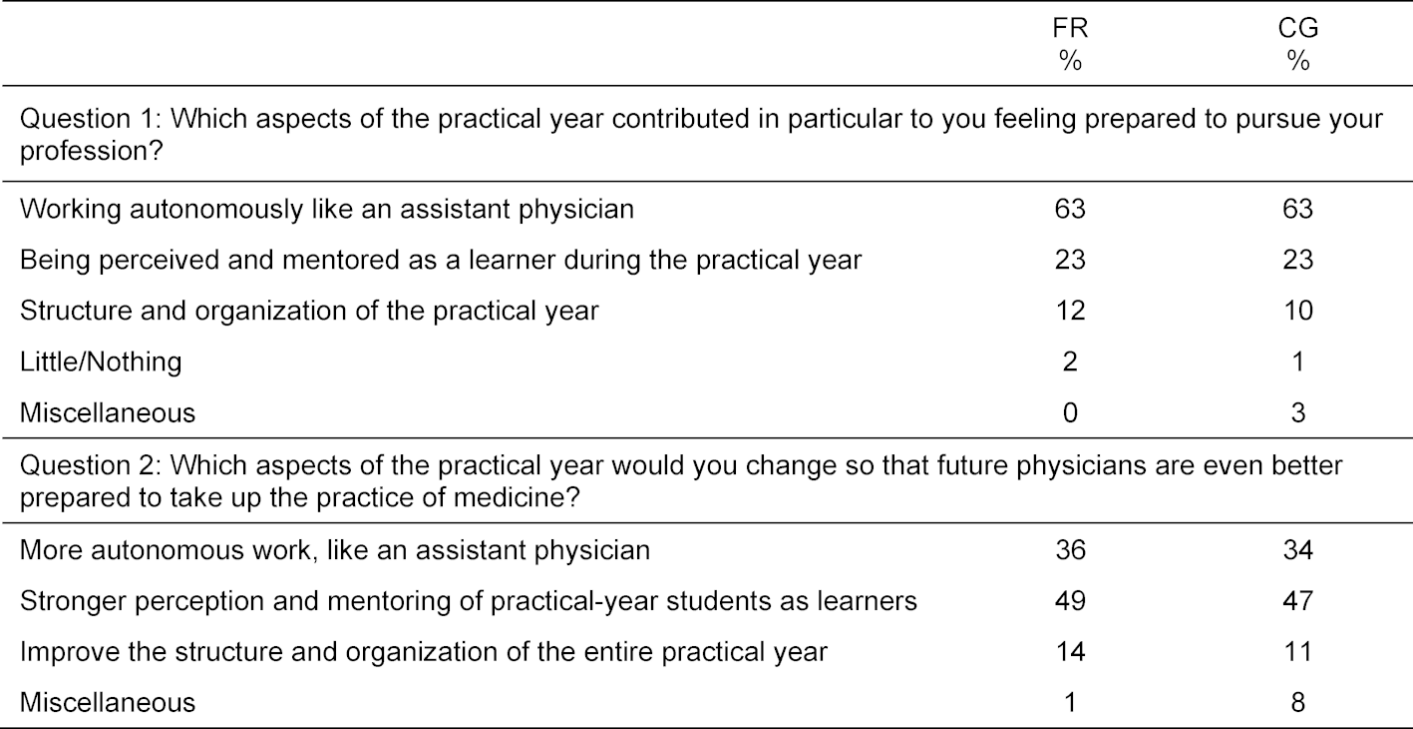
Main categories – percental distribution of the keyword mentioned for questions 1 and 2 for Freiburg (FR) and the other medical schools in Baden-Württemberg (comparison group = CG)
